# Letter to the Editor in Response to Zhou et al

**DOI:** 10.1093/infdis/jiab362

**Published:** 2021-07-12

**Authors:** Sean Troth, Joan Butterton, Carisa Stadlman DeAnda, Patricia Escobar, Jay Grobler, Daria Hazuda, George Painter

**Affiliations:** 1 Merck & Co, Kenilworth, New Jersey, USA; 2 Emory Institute of Drug Development, Emory University, Atlanta, Georgia, USA; 3 Drug Innovation Ventures at Emory, Atlanta, Georgia, USA; 4 Department of Pharmacology and Chemical Biology, Emory University, Atlanta, Georgia, USA

To the Editor—In their recent publication, Zhou et al [1] describe a concentration-dependent increase in rate of mutation in a modified in vitro Chinese hamster ovary cell hypoxanthine phosphorybosyl transferase assay with N-hydroxycytidine (NHC). NHC is the parent nucleoside of the 5’-isopropylester prodrug molnupiravir (MOV). In contrast, we have conducted a more comprehensive series of in vitro and in vivo genotoxicity studies, which, based on the totality of the data, demonstrate a low risk for genotoxicity with MOV in clinical use. We review these studies, as well as potential concerns with the methods used by Zhou et al.

While MOV and/or NHC have demonstrated the ability to induce mutations under specific in vitro culture conditions (including Ames and modified HPRT assays), extensive study of MOV in in vivo whole animal mutagenicity assays provides strong evidence of lack of in vivo relevance. Potential reasons for lack of translation of in vitro findings to in vivo mammalian systems may involve differences in metabolism, pharmacokinetics, exposure, replication, and DNA repair processes within a whole animal model compared with in vitro test conditions. It is well recognized that studies in appropriate in vivo models are needed to establish the biological significance and clinical risk of in vitro assay findings. As such, we conducted assays in 2 distinct rodent mutagenicity in vivo models that are recognized as robust tools for evaluating mutagenicity in vivo, and for assessing human risk for mutagenicity (Pig-a mutagenicity assay and Big Blue [cII locus] transgenic rodent assay] [2, 3]. 

In the Pig-a mutagenicity assay and Big Blue (cII locus) transgenic rodent assay, the impact of MOV treatment on mutation rates was not differentiable from mutation rates observed in untreated historical control animals. These in vivo mutation assays evaluated MOV at doses and durations significantly greater than those being used in the clinic. MOV was also negative for induction of chromosomal damage in in vitro micronucleus (with and without metabolic activation) and in vivo rat micronucleus assays. Thus, based on the totality of genotoxicity data (including 2 distinct in vivo rodent mutagenicity models in which MOV did not demonstrate evidence of mutagenicity or genotoxicity in vivo), MOV is considered to have low risk for genotoxicity in clinical use.

It is important to note that the assay conditions used by Zhou et al for their in vitro HPRT assay differed significantly from standard protocols conducted under regulatory test guidelines [4]. In the assay methods they describe [1, supplementary materials], several features make it difficult to interpret their results and compare them with existing published HPRT data. First, the cells were exposed continuously to NHC for a total of 32 days, substantially longer than the 3-6-hour exposure duration typically used per established guidelines [4]. Historical control data (used to determine performance of the assay in the laboratory with different positive and negative controls and to establish acceptable background mutant frequency ranges in untreated cells [5]) are not provided by the authors.

While NHC was shown to be toxic to CHO-K1 cells, when exposed at 10 µmol/L for 5 days [1, supplementary figure 4], cytotoxicity was not assessed at the end of the 32-day continuous exposure to NHC at ≤3 µmol/L. This step is needed to assess whether there was a reduction in relative survival of the treated cells compared with the control, to help differentiate direct test article–related mutagenicity versus mutations that may occur owing to DNA damage induced under cytotoxic conditions [6, 7].

The mutation results provided by Zhou et al were reported as total mutant colonies rather than mutant frequency [1], which does not allow for comparison of negative and positive control data to publicly available literature. The rationale for the NHC concentrations used in the assay (or concurrent control compounds) was not provided. To avoid potential false-positive results, the highest concentration tested should avoid producing precipitation in the culture media, marked changes in pH or osmolality, or excessive cytotoxicity [4]. Finally, information regarding the origin and purity of the NHC material used was not provided, and it is uncertain whether the stability or impurity of the material was characterized.

Given the state of the current coronavirus disease 2019 pandemic and the repeated and accelerating emergence of highly pathogenic coronaviruses, the development of potent antivirals with activity against severe acute respiratory syndrome coronavirus 2 and other coronaviruses is urgently needed. Our comprehensive safety evaluation coupled with the preclinical antiviral efficacy and clinical experience to date support the ongoing studies of MOV in patients, including those most likely to benefit from early intervention.

## Notes

***Acknowledgments.*** Dean P. Campbell, PhD, and Karyn Davis, BA, of Merck Sharp & Dohme, a subsidiary of Merck & Co, provided editorial support.

***Financial support.*** This work was supported by Merck Sharp & Dohme, a subsidiary of Merck & Co.

***Potential conflict of interests.*** S.T., J. B., C. S. D., P. E., J. G., and D. H. are employees of Merck Sharp & Dohme, a subsidiary of Merck & Co. and may own stock and/or stock options in Merck & Co. G. P. is an employee of Emory University and has a financial interest in molnupiravir. All authors have submitted the ICMJE Form for Disclosure of Potential Conflicts of Interest. Conflicts that the editors consider relevant to the content of the manuscript have been disclosed.
